# Cross-Feeding between Members of *Thauera* spp. and *Rhodococcus* spp. Drives Quinoline-Denitrifying Degradation in a Hypoxic Bioreactor

**DOI:** 10.1128/mSphere.00246-20

**Published:** 2020-04-29

**Authors:** Xinxin Wu, Xiaogang Wu, Ji Li, Qiaoyu Wu, Yiming Ma, Weikang Sui, Liping Zhao, Xiaojun Zhang

**Affiliations:** a State Key Laboratory of Microbial Metabolism, Shanghai Jiao Tong University, Shanghai, China; b Joint International Research Laboratory of Metabolic & Developmental Sciences, Shanghai Jiao Tong University, Shanghai, China; c School of Life Sciences & Biotechnology, Shanghai Jiao Tong University, Shanghai, China; National Institute of Advanced Industrial Science and Technology

**Keywords:** quinoline-degrading bioreactor, 2-hydroxyquinoline, microbial interaction, cross-feeding, oxygen, denitrification, biodegradation

## Abstract

The complex bacterial community in a quinoline-degrading denitrifying bioreactor is predominated by several taxa, such as *Thauera* and *Rhodococcus*. However, it remains unclear how the interactions between the different bacteria mediate quinoline metabolism under denitrifying conditions. In this study, we designed a sequence-specific amplification strategy to isolate the most predominant bacteria and obtained four strains of Thauera aminoaromatica, a representative of a key member in the bioreactor. Tests on these isolates demonstrated that all were unable to degrade quinoline but efficiently degraded 2-hydroxyquinoline, the hypothesized primary intermediate of quinoline catabolism, under nitrate-reducing conditions. However, another isolate, *Rhodococcus* sp. YF3, corresponding to the second most abundant taxon in the same bioreactor, was found to degrade quinoline via 2-hydroxyquinoline. The end products and removal rate of quinoline by isolate YF3 largely varied according to the quantity of available oxygen. Specifically, quinoline could be converted only to 2-hydroxyquinoline without further transformation under insufficient oxygen conditions, e.g., less than 0.5% initial oxygen in the vials. However, resting YF3 cells aerobically precultured in medium with quinoline could anaerobically convert quinoline to 2-hydroxyquinoline. A two-strain consortium constructed with isolates from *Thauera* (R2) and *Rhodococcus* (YF3) demonstrated efficient denitrifying degradation of quinoline. Thus, we experimentally verified that the metabolic interaction based on 2-hydroxyquinoline cross-feeding between two predominant bacteria constitutes the main quinoline degradation mechanism. This work uncovers the mechanism of quinoline removal by two cooperative bacterial species existing in denitrifying bioreactors.

**IMPORTANCE** We experimentally verified that the second most abundant taxon, *Rhodococcus*, played a role in degrading quinoline to 2-hydroxyquinoline, while the most abundant taxon, *Thauera*, degraded 2-hydroxyquinoline. Metabolites from *Thauera* further served to provide metabolites for *Rhodococcus*. Hence, an ecological guild composed of two isolates was assembled, revealing the different roles that keystone organisms play in the microbial community. This report, to the best of our knowledge, is the first on cross-feeding between the initial quinoline degrader and a second bacterium. Specifically, the quinoline degrader (*Rhodococcus*) did not benefit metabolically from quinoline degradation to 2-hydroxyquinoline but instead benefited from the metabolites produced by the second bacterium (*Thauera*) when *Thauera* degraded the 2-hydroxyquinoline. These results could be a significant step forward in the elucidation of the microbial mechanism underlying quinoline-denitrifying degradation.

## Abstract (precis)

We experimentally verified that the second most abundant taxon, *Rhodococcus*, played a role in degrading quinoline to 2-hydroxyquinoline, while the most abundant taxon, *Thauera*, degraded 2-hydroxyquinoline. Metabolites from *Thauera* further served to provide metabolites for *Rhodococcus*. Hence, an ecological guild composed of two isolates was assembled, revealing the different roles that keystone organisms play in the microbial community. This report, to the best of our knowledge, is the first on cross-feeding between the initial quinoline degrader and a second bacterium. Specifically, the quinoline degrader (*Rhodococcus*) did not benefit metabolically from quinoline degradation to 2-hydroxyquinoline but instead benefited from the metabolites produced by the second bacterium (*Thauera*) when *Thauera* degraded the 2-hydroxyquinoline. These results could be a significant step forward in the elucidation of the microbial mechanism underlying quinoline-denitrifying degradation.

## INTRODUCTION

Quinoline and its derivatives are typical N-heterocyclic compounds that occur widely in coal tar, shale oil, and creosote and serve as raw materials and solvents in the chemical, pharmaceutical, and pesticide industries (1). They are known to be carcinogenic and mutagenic to humans (2, 3) and have aroused significant concern as recalcitrant pollutants in the environment.

Anaerobic bioremediation is an attractive technology due to its energy savings and cost-effectiveness since heavily contaminated environments are often oxygen deficient (4). However, most of the literature has focused on the aerobic degradation of quinoline. Various microorganisms capable of metabolizing quinoline aerobically have been isolated, mostly belonging to *Pseudomonas* (5–7), *Rhodococcus* (8), and *Bacillus* (9). The pathways of aerobic quinoline degradation have also been well described (10). However, little attention has been paid to anaerobic quinoline biodegradation. The degradation of quinoline in industrial-scale anoxic wastewater (WW) treatment reactors has been reported in a few studies (11, 12). However, no evidence has proven the role of the main anaerobic degraders in these industrial bioreactors. There have been several efforts to identify the anaerobic degraders by using laboratory-scale bioreactors and batch culture experiments (13–16). However, to date, only one isolate, Desulfobacterium indolicum strain DSM 3383, which uses sulfate as an electron acceptor, obtained in pure culture has been shown to anaerobically degrade quinoline (17). It remains unknown why so few isolates that degrade quinoline under anaerobic conditions have been identified.

An efficient anoxic microbial community was enriched in a chemostat that was operated for more than 10 years with quinoline as the electron donor and nitrate as the electron acceptor. Phylogenetic analysis of this consortium showed that specific phylotypes were associated with different stages of degradation (16), which suggests that microorganisms interact during quinoline metabolism. However, our understanding of this interaction is restricted due to the complexity of the community composition in the bioreactor and the lack of anaerobic quinoline-degrading microorganisms available in pure culture. Therefore, functional analysis using representative isolates would be a crucial further step for understanding quinoline-denitrifying degradation in the reactor.

To investigate the underlying microbial processes in this complex quinoline-degrading consortium, we endeavored to isolate the most predominant and active bacteria, which were identified as the keystone organisms involved in denitrification and quinoline removal (16, 18). The degradation characteristics of these isolates were evaluated under different conditions. Based on the degradation function of different isolates, a coculture of representative isolates of *Thauera* and *Rhodococcus* was constructed to demonstrate the cooperation of two bacteria during quinoline metabolism under defined conditions.

## RESULTS

### Profiling of the quinoline-degrading bacterial community.

**FIG 1:**
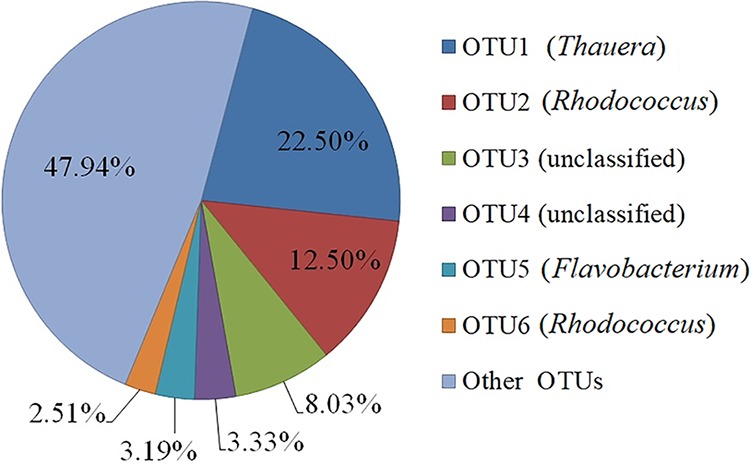
The abundance of the six most predominant OTUs among 460 OTUs from the quinoline-degrading bioreactor.

The removal rates of quinoline and nitrate remained stable at approximately 80% and 90%, respectively, in the denitrifying quinoline-degrading bioreactor. Prior to sample collection to isolate the most predominant organisms, the bacterial community in triplicate samples from the reactor was profiled by high-throughput sequencing. The Illumina MiSeq platform yielded 73,902 high-quality reads with an average of 24,634 reads per sample (±1,418 standard deviations [SD]). A total of 460 species-level operational taxonomic units (OTUs) were delineated using 97% identity as the cutoff value. Six predominant OTUs contributed to 52.06% of all reads (Fig. 1). The most predominant OTU (OTU1), with a relative abundance of 22.5%, belonged to the genus *Thauera*, while OTU2, the second most abundant OTU (12.5%), was affiliated with the genus *Rhodococcus.*

### Isolation and identification of the most predominant bacteria.

Since the most predominant OTU was assumed to belong to the key player in quinoline degradation, we attempted to isolate the corresponding bacteria from the bioreactor using a sequence-guided isolation strategy. Specific PCR primers based on the most predominant Thauera aminoaromatica isolate were designed *in silico*. Following selection based on the amplification, the specificity of the primer pair Thau135/Thau716 (5′-GGGATAACGTAGCGAAAGCT-3′ and 5′-CCATCGGTGTTCCTCCTG-3′) was further evaluated. The amplicon from the reactor sample generated using the selected primer pair was cloned, and 15 clones were randomly selected for partial sequencing of 16S rRNA genes with lengths greater than 500 bp. Among them, 8 clones showed 100% identity with DR-80 and the most predominant OTU1; 5 other clones shared over 99.7% identity with DR-80/OTU1; only 2 clones showed approximately 99.5% identity. These results indicated that the specificity of Thau135/Thau716 was sufficient for the subsequent isolation.

**FIG 2:**
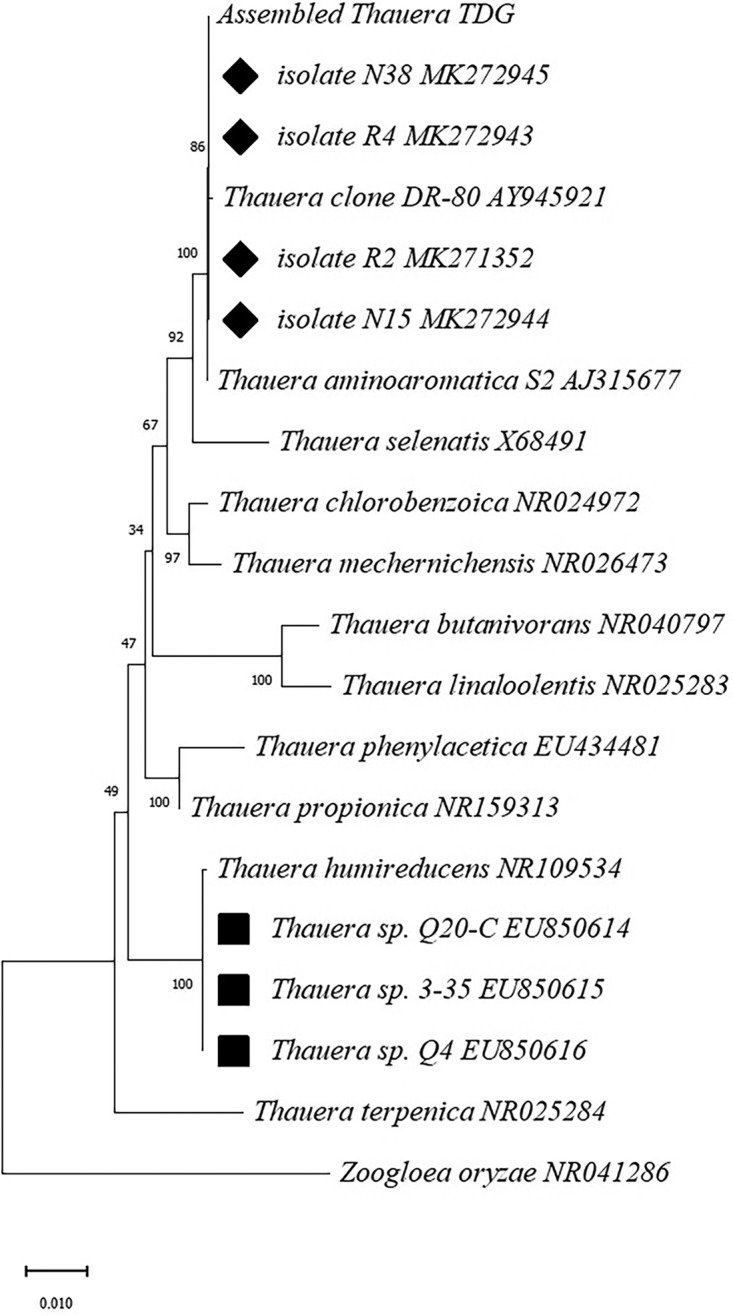
Phylogenetic tree based on the 16S rRNA gene sequences of isolates from quinoline-degrading bioreactors and other related *Thauera* species from the NCBI database. Numbers following the isolate names are the sequence accession numbers in GenBank. The bootstrap values of the neighbor-joining analysis are labeled at the nodes. Diamond labels indicate isolates analyzed in this study; square labels indicate isolates from the same reactor analyzed in a previous study.

Several hundred colonies grown on plates of six different types of medium were screened by specific amplification using the Thau135/Thau716 primers. Thirteen positive colonies were picked, of which 3 were purified from Reasoner’s 2A agar medium (R2A), 6 from 1/10-strength tryptic soy agar (TSA), and 4 from 1/10-strength nutrient agar (NA). Morphologically, these selected colonies were pearl-like on the 1/10 NA plate, and the cells formed characteristic flocs or clusters in NB liquid culture with shaking at 150 rpm and 30°C. All of these isolates were classified into four genotypes by the results of enterobacterial repetitive intergenic consensus PCR (ERIC-PCR) analysis, in which isolates R2, R4, N15, and N38 were selected as the representative strains for each ERIC type. The full-length 16S rRNA gene sequences of four representative strains were 100% identical to each other and had one nucleotide mismatch with the uncultured *Thauera* clone DR-80, which had the highest abundance in the laboratory-scale bioreactor for quinoline degradation (18). The full-length 16S rRNA gene sequences of four representative strains shared 100% identity with that from the most predominant *Thauera* genome, which was assembled using the metagenomics data for the same bioreactor in our previous work (19). Moreover, Thauera aminoaromatica strain S2 was found to be the closest neighbor of all isolates when the 16S rRNA gene sequences were subjected to a BLAST search against the NCBI nr database (Fig. 2), which is consistent with a previous report showing that the assembled genome of the most predominant organism in the reactor was most closely related to that of Thauera aminoaromatica S2 (30). These four isolates were phylogenetically differentiated from three other isolates, 3-35, Q4, and Q20-C, which were obtained previously in the quinoline-containing coking wastewater-denitrifying bioreactor from which the seeding sludge for our bioreactor was obtained (14).

### Metabolism of quinoline and 2-hydroxyquinoline by *Thauera* isolates.

**FIG 3:**
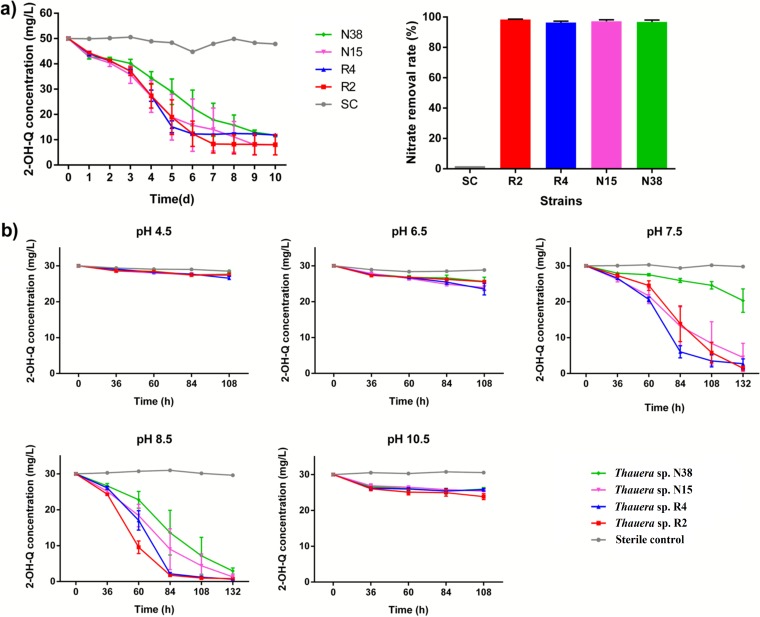
Dynamics of 2-hydroxyquinoline (2-OH-Q) concentrations (left) and nitrate removal (right) by four *Thauera* strains. (a) Degradation of 50 mg/liter 2-OH-Q and 1 mM nitrate. Time(d), time in days. (b) Degradation of 30 mg/liter 2-OH-Q under different pH conditions. SC, sterile control.

First, the isolated *Thauera* strains were tested for their capacity to degrade quinoline. Unexpectedly, none of them could metabolize quinoline when it was used as the sole carbon source, under both anaerobic and aerobic conditions. However, all four isolates could utilize 2-hydroxyquinoline, a derivative of quinoline, which is reported to be the first degradation intermediate of quinoline (20). The data showed that approximately 80% of 50 mg/liter 2-hydroxyquinoline was eliminated in MMHQ (mineral salt medium [MSM] supplemented with 0.23 mM 2-hydroxyquinoline) within 10 days under nitrate-reducing conditions and that there was no accumulation of intermediates in the measured samples (Fig. 3a). The effects of pH on 2-hydroxyquinoline degradation were further investigated. The results showed that 30 mg/liter 2-hydroxyquinoline was depleted in 132 h under both pH 7.5 and pH 8.5 conditions (Fig. 3b). The degradation of substrates was inhibited under acidic (pH ≤ 6.5) or alkaline (pH ≥ 10.5) conditions. The transformation of 2-hydroxyquinoline was pH dependent, with the optimal pH ranging from 7.5 to 8.5. Additionally, although all of the isolates had the capacity for 2-hydroxyquinoline removal, their degradation rates were different. For example, isolate N38 had the lowest rate at 0.228 mg/liter/h, whereas isolate R2 had the highest rate at 0.336 mg/liter/h.

### Metabolism of quinoline by a *Rhodococcus* isolate.

**FIG 4:**
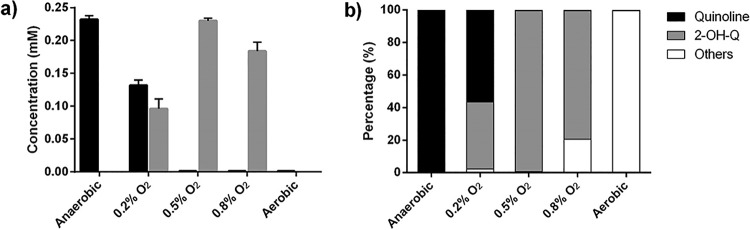
Transformation of quinoline by *Rhodococcus* sp. YF3 under different oxygen availability conditions. (a) Concentrations of quinoline and 2-hydroxyquinoline (2-OH-Q). (b) Proportions of different components remaining in the cultures.

On the petri dish used for bacterial isolation, a type of reddish colony frequently appeared. Identification via 16S rRNA gene sequencing suggested that it represented another predominant bacterial phylotype for quinoline degradation proposed in a previous study (16). The isolate YF3 was selected as the representative of the most predominant *Rhodococcus* OTU, which showed the closest identity with Rhodococcus pyridinivorans. The transformation of quinoline by YF3 with different amounts of initial oxygen in the headspace of vials was explored. We found that the quinoline removal rates and the results of transformation of products differed due to the different oxygen supplementation conditions. After 24 h of incubation, the degradation mediated by microorganisms included in the inoculum grown on NB medium was analyzed. The results showed that 0.23 mM quinoline was completely oxidized when the medium was supplemented with sufficient oxygen; in contrast, quinoline persisted under anaerobic (with nitrate supplementation) conditions, whereas approximately 0.09 mM 2-hydroxyquinoline accumulated and 0.13 mM quinoline remained in the presence of 0.2% oxygen, which is equal to 2.8 mg/liter dissolved oxygen. Quinoline was completely transformed into 2-hydroxyquinoline without further transformation under conditions of 0.5% oxygen, which is equal to 7.1 mg/liter dissolved oxygen, and under conditions in which the oxygen concentration increased to 0.8%, which is equal to 11.2 mg/liter dissolved oxygen, only 0.17 mM 2-hydroxyquinoline remained due to further degradation (Fig. 4).

### Synergistic degradation of quinoline by a bacterial coculture.

**FIG 5:**
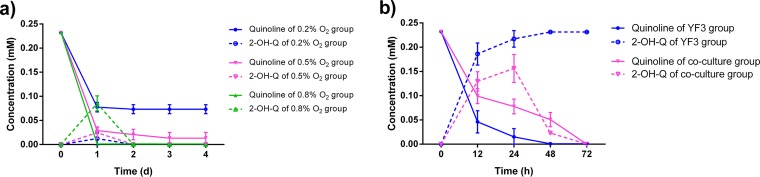
Dynamics of quinoline degradation by the coculture of *Rhodococcus* sp. YF3 and *Thauera* sp. R2. (a) Limited-oxygen-supplementation conditions with the inoculum cultured in NB medium; the different colors represent different oxygen supply conditions. (b) Anaerobic conditions with the inoculum precultured aerobically in medium containing quinoline. The blue line represents *Rhodococcus* sp. YF3 alone, whereas the red line represents the coculture of *Rhodococcus* sp. YF3 and *Thauera* sp. R2. Solid lines in panels a and b represent the changes in the quinoline concentrations, whereas the dotted lines represent the changes in the 2-hydroxyquinoline (2-OH-Q) concentrations.

Since *Thauera* sp. R2 was found to be an anaerobic 2-hydroxyquinoline degrader and since *Rhodococcus* sp. YF3 has the capacity to hydroxylate quinoline, we hypothesized that the degradation of quinoline might be accomplished in the bioreactor via metabolic cooperation of these two bacteria. Thus, we performed a cocultivation trial using the bacterial strains of *Thauera* and *Rhodococcus* mentioned above to test their quinoline degradation capacity under different oxygen availability conditions. We found that 0.07 mM and 0.01 mM quinoline remained in the vials of the 0.2% O_2_ and 0.5% O_2_ experimental groups, respectively. 2-Hydroxyquinoline was finally eliminated in all the groups within 48 h, while quinoline was completely depleted in only the 0.8% O_2_ group (Fig. 5a).

Considering that the original bioreactor represented an oxygen-deficient system, we attempted to elucidate the possible mechanism of anaerobic quinoline degradation. We verified that *Rhodococcus* sp. YF3 could hydroxylate quinoline under micro-oxygen conditions. We washed and transferred the YF3 cells, which were preincubated with quinoline, 2-hydroxyquinoline, or phenol for 24 h under 0.5% oxygen conditions, to fresh MMQ (MSM supplemented with 0.23 mM quinoline) and incubated them for another 72 h under completely anaerobic conditions and found that 0.23 mM quinoline was transformed into equivalent 2-hydroxyquinoline by the YF3 monoculture. 2-Hydroxyquinoline was further removed in the YF3 and R2 coculture (Fig. 5b).

## DISCUSSION

In this study, we aimed to elucidate the microbial mechanism of quinoline-denitrifying degradation. As a first step, we sought to isolate the most predominant bacterial species in the quinoline-degrading consortium, which we considered to be a promising anaerobic quinoline degrader. However, many attempts performed using media with quinoline as the sole carbon source have failed to isolate the target strain. In an earlier study, a *Thauera* genus-specific nested-PCR denaturing gradient gel electrophoresis (DGGE) method was developed and was combined with the use of media containing various carbon sources to facilitate the isolation of *Thauera* spp. In that study, three *Thauera* strains (Q4, Q20-C, and 3-35) were obtained, but none of them could utilize quinoline either aerobically or anaerobically (13, 14). Considering that the former isolates corresponded to the less abundant *Thauera* species in the bioreactor, in this study, we designed OTU-specific PCR primers targeting the most predominant *Thauera* OTU1 as a biomarker to guide the isolation of the target *Thauera* bacterial strains. The colonies that grew on various media were screened by using this method. As a result, four isolates belonging to *Thauera* genus were identified as homologs of the most predominant bacteria in the quinoline-degrading bioreactor. Thauera aminoaromatica has been reported to be a denitrifying species capable of growing in the presence of aromatic-amino compounds (21). In this study, all isolates with 16S rRNA gene sequence identity with the most predominant *Thauera* OTU in the bioreactor were able to degrade 2-hydroxyquinoline but not quinoline under denitrifying conditions, which precisely explains the previous unsuccessful attempts to isolate quinoline-degrading bacteria by using quinoline as the sole carbon source. Hence, a sequence-guided approach to isolate difficult-to-culture bacteria could be an alternative and promising method.

Dozens of studies have reported that isolated bacteria may lack the ability to completely mineralize hydrocarbons, especially for recalcitrant compounds (22). Moreover, regardless of the presence or absence of oxygen, the first metabolite of quinoline is 2-hydroxyquinoline (10). This compound was thus preferentially considered a substrate of the most predominant *Thauera* bacteria in the denitrifying bioreactor. The results showing 2-hydroxyquinoline degradation by the monoculture of *Thauera* isolates in this study proved our hypothesis.

To further identify the bacteria responsible for transforming quinoline to 2-hydroxyquinoline, we further explored other key players among the predominant bacteria in the community. The members of the genus *Rhodococcus* are known to be aerobic quinoline degraders (8), and this phylotype has been reported to be closely associated with quinoline-denitrifying degradation (15, 16). Consequently, *Rhodococcus* sp. YF3 isolated from the bioreactor was considered a candidate to initially degrade quinoline in the denitrifying bioreactor. Unsurprisingly, the experimental result was consistent with our hypothesis. Notably, the consumption of quinoline by strain YF3 depended on the oxygen concentration. But our result proved that *Rhodococcus* has no ability to degrade 2-hydroxyquinoline under denitrifying condition. Therefore, long-term access to low levels of oxygen likely shapes the *Rhodococcus*-dominated community in the quinoline-denitrifying bioreactor (15).

**FIG 6:**
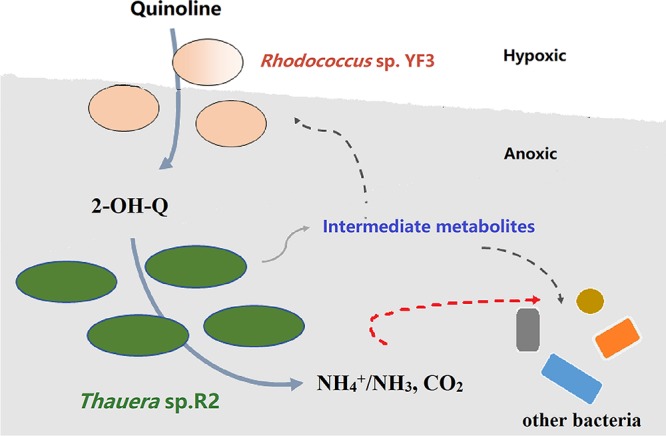
Proposed model for the syntrophic degradation of quinoline by *Rhodococcus* sp. YF3 and *Thauera* sp. R2.

Communities of bacteria are extraordinarily complex, with hundreds of interacting taxa, but knowledge about how the tangled interactions within natural bacterial communities mediate ecosystem functioning is limited (23). A promising way to surpass this limitation is to create a synthetic community by artificially coculturing two or more selected species under well-defined conditions (24). Considering that the metabolite of quinoline transformed by isolate YF3 could be further metabolized by isolate R2, we constructed a synthetic consortium using pure cultures of these two isolates to achieve the cooperative degradation of quinoline. In this consortium, *Thauera* sp. R2 cross-fed on the 2-hydroxyquinoline released by *Rhodococcus* sp. YF3. Ecologically, this type of microbial interaction is equivalent to commensalism (25). Here, the initial step of quinoline degradation is hydroxylation, which requires energy (26). Accordingly, *Rhodococcus* sp. YF3 does not benefit from the hydroxylation reaction. In this study, the product of the first 2-hydroxyquinoline transformation step served as the substrate for the next organism in the chain. Furthermore, *Thauera* sp. R2 released certain intermediates during further metabolism. *Rhodococcus* sp. YF3 could utilize the metabolites released by *Thauera* cells to obtain energy and grow. Consequently, most of the carbon from quinoline was consumed by isolate R2 cells and a small proportion by isolate YF3 and other bacteria. This result is consistent with the high abundance of *Thauera* sp. R2 and relatively low abundance of *Rhodococcus* sp. YF3 in the bioreactor, although the latter plays a fundamental role in degrading quinoline to provide the 2-hydroxyquinoline required by *Thauera* sp. R2. Thus, the cross-feeding and interdependence of these two strains are the main driving forces for quinoline degradation and assembly by a stable community in the hypoxic-anoxic bioreactor. We summarize the ecological mechanism of quinoline degradation in Fig. 6.

Moreover, it is worth considering why this multistrain degradation occurred in the quinoline-degrading reactor. In previous research (27), the main reactions for starch biodegradation occurred in the lower part of the reactor, while the small molecules such as acetate were converted to methane at the upper part. The organic compounds were gradually reduced along the upflow direction. We speculated that in our closed reactor, during synthetic wastewater upflow (i.e., from the bottom to the top), the dissolved oxygen in the synthetic wastewater (ca. 7 mg/liter dissolved oxygen) is rapidly consumed by *Rhodococcus* and other aerobic bacteria in the bottom of the reactor, which then produces an anoxic denitrifying niche in the inner space of the bioreactor for *Thauera* to degrade the 2-hydroxyquinoline produced via hydroxylation by *Rhodococcus* activity under limited-oxygen or anoxic conditions.

Metabolic cooperation between/among different bacterial members in the community is beneficial to competitiveness. For instance, it has been shown that the degradation of 4-chlorodibenzofuran by *Sphingomonas* sp. RW1 results in the accumulation of the dead-end product 3,5-dichlorosalicylate, whereas inoculation with *Burkholderia* sp. JWS enables complete degradation via cooperation (28). A consortium composed of Escherichia coli SD2 and Pseudomonas putida KT2440 pSB337 was previously shown to degrade parathion efficiently without the accumulation of toxic intermediates (29). In addition, it has been reported that four members are actively involved in the degradation of 4-chlorosalicylate, in which the strain of MT4 alleviates the stress of MT1 by transforming the toxic intermediate protoanemonin (30). These examples indicate that the metabolic complementarity in the microbial community favors the efficient bioremediation of contaminants and benefits the adaptation or survival of bacteria in the specific environment.

Taking the data together, this report has collectively discussed commensal interactions in a quinoline-degrading consortium, aiming to highlight the importance of cross-feeding relationships between/among bacteria involved in the biodegradation of organic compounds in the environment. Although our knowledge of this commensal relationship is not yet complete, understanding such complex microbial interactions could provide useful information for assessing the biodegradability of organic compounds in the natural environment, for instance, quinoline-denitrifying degradation. Here, we provide unexpected and notable insight into microbial interactions. *Thauera* sp. R2, which adapts to the denitrifying niche, can cooperate with *Rhodococc*us sp. YF3 to form a cross-feeding community. To the best of our knowledge, this is the first report of a cooperative relationship in quinoline-denitrifying degrading communities, where two cross-feeding bacterial strains cooperate to degrade quinoline. However, the key bacteria (*Rhodococcus*), which is required to degrade quinoline to 2-hydroxyquinoline, did not directly benefit from hydroxylation under limited-oxygen conditions.

## MATERIALS AND METHODS

### Operation of the quinoline-denitrifying bioreactor.

A 2-liter tank filled with plastic rings and synthetic fiber strings as semisoft media was used to construct a laboratory-scale bioreactor. Seeding sludge was collected from an anoxic tank of a coking wastewater treatment plant from the Shanghai Coking and Chemical Factory (Wujing, Shanghai). The synthetic wastewater was composed of quinoline (100 mg/liter), NaNO_3_ (240 mg/liter), and K_2_HPO_4_ (140 mg/liter). During wastewater upflow (i.e., from the bottom to the top) without any agitation, the inner part of the reactor was depleted of oxygen due to its rapid consumption by aerobic bacteria. Thus, the reactor was hypoxic to anoxic from the influent to the effluent. The hydraulic retention time was 24 h. The pH and temperature of the reactor were adjusted and maintained at 7.5 and 25°C, respectively.

### Profiling of the quinoline-degrading bacterial community.

The biofilm sample was collected by scraping the biofilm from the surface of the supporting materials in the bioreactor. Genomic DNA extraction of the samples was conducted in triplicate as previously described (31). The sequencing library of the V3-V4 regions in the 16S rRNA gene was constructed by two-step PCR amplification according to Illumina’s instructions. The purified amplicons were sequenced using the Illumina MiSeq System (Illumina Inc., USA). The preliminary processing of the raw sequencing data was conducted as previously described (32). Sequence assembly was implemented first; the unique sequences obtained by dereplication were sorted by decreasing abundance, and the singletons were discarded. UPARSE defaults (33) were used to select the representative operational taxonomic units (OTUs), and UCHIME (34) was selected to perform further reference-based chimera detection against the RDP classifier training database (35). Finally, the OTU table was completed by mapping quality-filtered reads to the representative OTUs with Usearch (36), resulting in a global alignment algorithm with a 97% cutoff. Further analysis was performed using the QIIME platform (version 1.8) (37). In addition, sequences representative of each OTU were submitted to the online RDP classifier (RDP database version 2.11) to determine the phylogeny, with a bootstrap cutoff of 80%. The 16S rRNA gene sequences in this study were submitted to the GenBank Sequence Read Archive (SRA) database at the National Center for Biotechnology Information (NCBI) under accession number SRP188486.

### Isolation and identification of the most predominant bacteria.

Six types of media were used to isolate the most predominant Thauera aminoaromatica strain. The compositions of the six media were as follows: for nutrient agar (NA), peptone 10 g/liter, beef extract 3 g/liter, NaCl 5 g/liter, agar 15 g/liter (38); for 1/10-strength NA, 10-fold dilution based on NA except for the content of agar; for tryptic soy agar (TSA), tryptone 17 g/liter, soy peptone 3 g/liter, glucose 2.5 g/liter, NaCl 5 g/liter, K_2_HPO_4_ 2.5 g/liter, agar 15 g/liter (39); for 1/10-strength TSA, 10-fold dilution based on TSA except for the content of agar; for Reasoner’s 2A agar (R2A), yeast extract 0.5 g/liter, peptone 0.5 g/liter, casein hydrolysate 0.5 g/liter, glucose 0.5 g/liter, soluble starch 0.5 g/liter, KH_2_PO_4_ 0.3 g/liter, MgSO_4_ 0.024 g/liter, sodium pyruvate 0.3 g/liter, agar 15 g/liter (39); for wastewater medium (WWM), raw wastewater subjected to filtration and removal of bacteria and supplemented with 1.5% agar.

The specific PCR primers targeting the most predominant Thauera aminoaromatica bacteria were designed by the use of DNAMAN (version 7.0) with the sequences of different OTUs belonging to the genus *Thauera*, which were obtained from a full-length 16S rRNA clone library constructed for a quinoline-denitrifying bioreactor (18). OTU-specific primers were used to assist the screening of the target *Thauera* strains by following a previously described procedure (13).

The biofilm sample was obtained and mixed in a shaker at 150 rpm for 2 h. Then, the suspension was diluted and spread on the plates of six types of media, including NA, 1/10-strength NA, TSA, 1/10-strength TSA, R_2_A, and WWM. All plates were incubated at 30°C under aerobic and anaerobic conditions. The isolates that had positive colony PCR signals were purified by plate-streaking technology. Finally, the genomic DNA of all isolates was extracted by the phenol-chloroform protocol (40). ERIC-PCR was conducted for the genotyping of all isolates (41). Representative strains for different types of ERIC fingerprints were selected for 16S rRNA gene sequencing to identify the taxonomy and construct a phylogenetic tree.

### Preculture of bacterial inoculum for the biodegradation experiment.

Unless specifically mentioned otherwise, the inoculum for all experiments was prepared by inoculating the isolated representative strains of *Thauera* spp. or *Rhodococcus* sp. YF3 (GU143680.1) in nutrient broth medium (NB) and incubation at 30°C at 150 rpm on a rotary shaker for 24 h. The bacterial cells were harvested by centrifugation and washed three times in mineral salt medium (MSM), and the suspension was used as an inoculum. The mineral salt medium (MSM) contained the following ingredients: K_2_HPO_4_·3H_2_O 0.57 g/liter, KH_2_PO_4_ 0.233 g/liter, NH_4_Cl 0.02675 g/liter, NaCl 0.5 g/liter, and NaNO_3_ 85 mg/liter (1 mM), with trace elements, including NaHCO_3_ 0.168 g/liter, MgSO_4_ 0.12 g/liter, CaCl_2_ 0.0544 g/liter, disodium EDTA 0.025 g/liter, H_3_BO_3_ 0.0036 g/liter, FeSO_4_·7H_2_O 0.0015 g/liter, CoCl_2_ 0.0012 g/liter, Ni(NH_4_)_2_(SO_4_)_2_ 0.0012 g/liter, Na_2_MoO_4_ 0.00094 g/liter, Na_2_SeO_4_ 0.00026 g/liter, MnSO_4_ 0.0002 g/liter, ZnSO_4_ 0.00016 g/liter, and CuSO_4_ 0.000032 g/liter, pH 7.4.

### Degradation experiments for bacterial isolates.

The batch experiments were conducted using a series of 20 ml headspace vials, which contained 10 ml of either MMQ or MMHQ. The compositions of MMQ and MMHQ were as follows. MMQ, mineral salt medium (MSM) supplemented with 0.23 mM quinoline (Sigma-Aldrich, Co., Inc.); MMHQ, mineral salt medium (MSM) supplemented with 0.23 mM 2-hydroxyquinoline (Sigma-Aldrich, Co., Inc.).

The inoculum was 5% to 10% of the volumetric proportion. For each of the different media, vials without inoculum but maintained under the same conditions were set as the abiotic negative controls. All vials were immediately sealed with airtight butyl rubber septa and aluminum crimp caps and made anoxic by repeated evacuation and filling with helium. To investigate the effects of pH on degradation, the initial pH of the media was adjusted to 4.5, 6.5, 7.5, 8.5, and 10.5.

To investigate the effects of oxygen on quinoline biotransformation by *Rhodococcus* sp. YF3, 0.04 ml, 0.10 ml, and 0.16 ml of pure oxygen, named groups 0.2% O_2_, 0.5% O_2_, and 0.8% O_2_, respectively, were injected into the vials by the use of precision syringes. The headspace of the anaerobic vials was not injected with oxygen, whereas the headspace of the aerobic vials was replaced with air. All vials were incubated in the dark at 30°C with shaking at 150 rpm. Samples were taken periodically by syringe in an anaerobic workstation to exclude oxygen during incubation. All treatments in the degradation experiment described above were performed in triplicate to minimize the experimental variation.

### Measurement of chemical compounds.

The sampled liquid was centrifuged, and the supernatant was used for further analysis. The concentrations of quinoline and 2-hydroxyquinoline were analyzed by the use of a high-pressure liquid chromatography (HPLC) system (Agilent; Zorbax SB-C_18_ reverse-phase column, 150 by 4.6 mm, 5-μm pore size). The mobile phase was a methanol solution with a volume ratio of 60:40 (methanol/water) used at a flow rate of 1.0 ml/min. Quinoline and 2-hydroxyquinoline were both detected at a 225-nm wavelength. The injection volume was 20 μl, and the column temperature was 30°C. The nitrate concentration was measured using a PXJ-1B ion meter (Jiangfen, China) with a pNO_3_^−1^ nitrate ion-selective electrode (Tianci, Shanghai).

### Data accessibility.

The 16S rRNA gene sequences of representative strains for different types of ERIC fingerprints were deposited in GenBank under accession numbers MK271352 (R2), MK272943 (R4), MK272944 (N15), and MK272945 (N38).
